# Pitfalls of diagnosing pituitary hypoplasia in the patients with short stature

**DOI:** 10.1007/s12020-024-03951-9

**Published:** 2024-07-05

**Authors:** Seniha Kiremitci Yilmaz, Gülgün Yilmaz Ovali, Deniz Ozalp Kizilay, Serdar Tarhan, Betul Ersoy

**Affiliations:** 1 Division of Pediatric Endocrinology, Health Sciences University, Istanbul Haseki Training and Research Hospital, Istanbul, Turkey; 2 Department of Radiology, Celal Bayar University, Faculty of Medicine, Manisa, Turkey; 3 Division of Pediatric Endocrinology and Metabolism, Ege University, Faculty of Medicine, İzmir, Turkey; 4 Division of Pediatric Endocrinology and Metabolism, Celal Bayar University, Faculty of Medicine, Manisa, Turkey

**Keywords:** Pituitary volume, Pituitary hypoplasia, Bone age, Short stature, Recombinant GH response, Multiple pituitary hormone deficiency

## Abstract

**Purpose:**

Height age (HA) and bone age (BA) delay is well known in the patients with short stature. Therefore assessing pituitary hypoplasia based on chronological age (CA) might cause overdiagnosis of pituitary hypoplasia. We aimed to investigate the diagnostic and prognostic value of the PH and PV based on CA, HA, or BA in the patients with GHD.

**Methods:**

Fifty-seven patients with severe and 40 patients with partial GHD and 39 patients with ISS assigned to the study. For defining the most accurate diagnosis of pituitary hypoplasia, PH and PV were evaluated based on CA, BA and HA. The relationship of each method with clinical features was examined.

**Results:**

The mean PV was significantly larger in patients with ISS compared to the GH-deficient patients. PV was more correlated with clinical features including height SDS, stimulated GH concentration, IGF-1 and IGFBP-3 SDS, height velocity before and after rGH therapy. We found BA-based PV could discriminate GHD from ISS (Sensitivity: 17%, specificity: 98%, positive predictive value: 94%, negative predictive value: 39%), compared to the other methods based on PH or PV respect to CA and HA. 3% of patients with ISS, 17% of patients with GHD had pituitary hypoplasia based on PV-BA.

**Conclusion:**

PV based on BA, has the most accurate diagnostic value for defining pituitary hypoplasia. But it should be kept in mind that there might be still misdiagnosed patients by this method. PV is also a significant predictor for the rGH response.

## Introduction

Pituitary gland (PG) imaging is performed in patients diagnosed with growth hormone (GH) deficiency (GHD), to ascertain cause of GHD. Pituitary hypoplasia is the most common PG abnormalities. Pituitary hypoplasia rate was reported as 7.8% [1]. Several studies have reported that pituitary hypoplasia might be useful for diagnosing and predicting prognosis of GHD [2–4]. So establishing the most accurate diagnosis of pituitary hypoplasia is important. Previously PG measurements were focused on pituitary height (PH) [5]. Subsequently researchers suggested that the exact PG size did not correlate with one dimension, because of the various morphologies of normal PG [6].

Pituitary hypoplasia is traditionally diagnosed by evaluating PG size according to chronological age (CA). Nevertheless short children, diagnosed with idiopathic short stature (ISS) or GHD have frequently height age (HA) or bone age (BA) delay. So a new question is appeared: if the patients’ CA are different from their HA and BA, might it be clinically misleading to assess size of PG only based on CA? To best of our knowledge there is still no exact data for defining pituitary hypoplasia, and comparing definitions by different basis of evaluation for pituitary hypoplasia. In this study we aimed to clarify the most reliable diagnostic method to determine pituitary hypoplasia in short children and investigate the association of PG size with response to recombinant GH (rGH) therapy and developing multiple pituitary hormon deficiencies (MPHD).

## Materials and methods

This retrospective study was conducted on 97 children with GHD and 39 children with ISS. The patients’ data was collected from medical records and Picture Archiving Communication Systems for pituitary MRI. Children with any chronic diseases or other endocrine abnormalities, and genetic syndrome, MPHD at presentation, evidence of hypothalamo-pituitary lesions, prematurity and/or small for gestational age birth were excluded. The study was approved by local ethics committee (Celal Bayar University, number: 20478486-232).

The main criteria for diagnosis of GHD had been: height was more than 2 SD below the corresponding mean height according to CA, sex and national data and height velocity (HV) at 6-month follow-up more than 1 SD below mean CA and stimulated GH less than 10 ng/mL. ISS was defined as height was more than 2 SD below the corresponding mean height according to CA, sex and national data and stimulated GH more than 10 ng/mL. Both insuline induced hypoglycemia and L-Dopa stimulation tests were performed after an overnight fasting. Hypoglycemia (blood glucose < 2.2 mmol/L) achieved in all cases during insuline induced hypoglycemia test. The stimulated GH was defined as highest GH concentration obtained from tests. According to stimulated GH, the patients were classified as having severe GHD if GH was below 7 ng/mL, partial GHD if GH was between 7 and 10 ng/mL, or ISS if GH was above 10 ng/mL. Normal cortisol response was determined if cortisol was more than 18 µg/dL. Serum GH, IGF-1, and IGFBP-3 concentrations were measured by a chemiluminescence assay on Immulite 2000 autoanalyser (DPC, Flanders, NJ, USA) that had intra and inter-assay CVs of 3.0% and 6.2%, respectively. IGF-1 SDS and IGFBP-3 SDS were calculated according to BA by using electronic databases (https://www.ceddcozum.com/). Physical examination findings were obtained at the time of the stimulation tests. Since PG morphology may change throughout childhood, we also evaluated prepubertal and pubertal children separately [7].

All patients with GHD had undergone pituitary neuroimaging within two months after tests. Same experienced neuroradiologist that blinded to clinical features, performed assessments of images. All images had been performed with 1.5Tesla MRI scanner (SignaHDx, General-Electric-Healtcare, Wisconsin, USA). Images were obtained by using 2 mm slice thickness, 256 × 256 matrix. T1/SE coronal/sagittal, T2(SE/TSE) coronal and dynamic contrasted TSE/T1 coronal and post conrast SE/T1 coronal/sagittal images were performed. None of the patients had pituitary stalk abnormalities. Upper border of PG was evaluated on midsagittal section. PH was measured as greatest distance between upper and lower borders of PG on coronal, and sagittal planes. Since PH was measured on coronal plane in Turkish data we compared, we evaluated PH on coronal plane. PH was similar on both planes (p = 0.2). Pituitary width and lenght were measured longest horizontal diameter on coronal plane and longest anteroposterior diameter on sagittal plane. PV was calculated by using formula (0.52 x height x width x lenght). Pituitary hypoplasia was designated as a PH or PV 2 SD below compared with normal age and sex matched Turkish children [8]. Pituitary measurements were evaluated according to not only CA but also HA and BA. BA was estimated with left hand/wrist radiograph by the same endocrinologist by using Greulich-Pyle atlas.

All patients with GHD had rGH treatment after pituitary neuroimaging. HV and height SD gain after rGH therapy were calculated. Response to rGH and development of MPHD were evaluated to determine the prognostic effects of PG size. Good rGH response was defined as above 0.3 SD per year in height SD gain [9]. The relationship between clinical/biochemical characteristics and all pituitary measurements were examined.

All patients with GHD had undergone evaluation for MPHD at the time of diagnosis and annually. Serum free-thyroxine, thyroid stimulating hormone (TSH), adrenocorticotropic hormone (ACTH), cortisol were performed using an immunchemiluminescence assay. When morning cortisol was detected below 5 µg/dL, low dose ACTH stimulation test (1 µg) was performed. If stimulated cortisol was below 18 µg/dL, adrenal insufficiency was diagnosed.

### Statistical analysis

Statistical analyses were performed using SPSS software (IBM SPSS Statistics 22). Categorical variables were compared using Chi square test. Comparison of categorical variables obtained from same patients according to different diagnostic methods was made with Mc Nemar test. To compare continuous variables student’s t and Mann-Whitney-u tests were used. Correlations were tested by Pearson’s and Spearman rank correlation coefficients. For more than two independent groups comparison, ANOVA and Kruskall Wallis tests were preferred. Multiple regression analysis including CA, BA, height SDS, HV, gender, pubertal staging, stimulated GH, IGF-1 SDS, PH and PV were performed, when investigating factors effects rGH response and developing MPHD. We performed binary logistic regression analysis including dependent variables (PV-CA, PV-HA, PV-BA and PH-CA, PH-HA, PH-BA) and independent variables (CA, HA, BA, pubertal stage, gender, height SDS, height velocity, stimulated GH, IGF-1 SDS, and IGFBP-3 SDS) to investigate effects of the clinical/biochemical features on pituitary size. A p value < 0.05 was considered statistically significant.

## Results

Fifty seven patients had severe GHD, with the mean age of 11.5( ± 2.7) years. 40 patients with the mean age of 11.6( ± 2.4) years had partial GHD, and 39 patients with the mean age of 12.3( ± 2.4) years were diagnosed as ISS. The mean CA-HA and CA-BA differences were 3( ± 1) and 2.5( ± 1.4) years in severe GHD, 2.8( ± 1) and 2.1( ± 1.4) years in partial GHD and 3.2( ± 0.9) and 2.2( ± 1.5) years in ISS groups, (p > 0.3). All clinical features were presented in Table 1. All patients with GHD had been treated with rGH with mean dose of 31 ± 5 µg/kg/d, at least 2 years.

**Table 1:** Baseline features of the patients with growth hormone deficiency and idiopathic short stature

	Severe GHD (*n* = 57)	Partial GHD (*n* = 40)	ISS (*n* = 39)	*p*-value
Demographic characteristics				
Age (years)	11.4 ± 2.7	11.5 ± 2.4	12.3 ± 2.4	*p* = 0.08
Height age (years)	8.4 ± 2.4	8.6 ± 2	9 ± 2	*p* = 0.2
Bone age (years)	8.9 ± 3	9.4 ± 2.5	10 ± 2.7	*p* = 0.06
Sex (F/M)	26/31	23/17	16/23	0.6
Prepubertal/pubertal	29/28	17/23	12/27	0.08
Birth weight (g)	3200 ± 500	3200 ± 350	3500 ± 600	*p* = 0.07
Clinical characteristics				
Height SDS	−2.7 ± 0.6	−2.6 ± 0.9	−2.7 ± 0.7	*p* = 0.8
BMI SDS	0.1 ± 1.4	−0.4 ± 1	−0.6 ± 1	*p* = 0.03#
Height velocity (cm/year)	5 ± 1.6	5 ± 2.4	5.2 ± 1.6	*p* = 0.3
IGF-1 SDS	−1.4 ± 1	−1.4 ± 1.4	−1.3 ± 1.4	*p* *=* *0.7*
IGFBP-3 SDS	−0.2 ± 1.9	−0.8 ± 1.5	−0.06 ± 1.4	*p* = 0.1

*BMI* Body mass index, *GHD* Growth hormone deficiency, *IGF-1* Insuline like growth factor 1, *IGFBP-3* Insüline like growth factor binding protein 3, *ISS* Idiopathic short stature, *SDS* Standard deviation score

^#^BMI SDS was significantly different between severe GHD and ISS groups

**Fig. 1:**
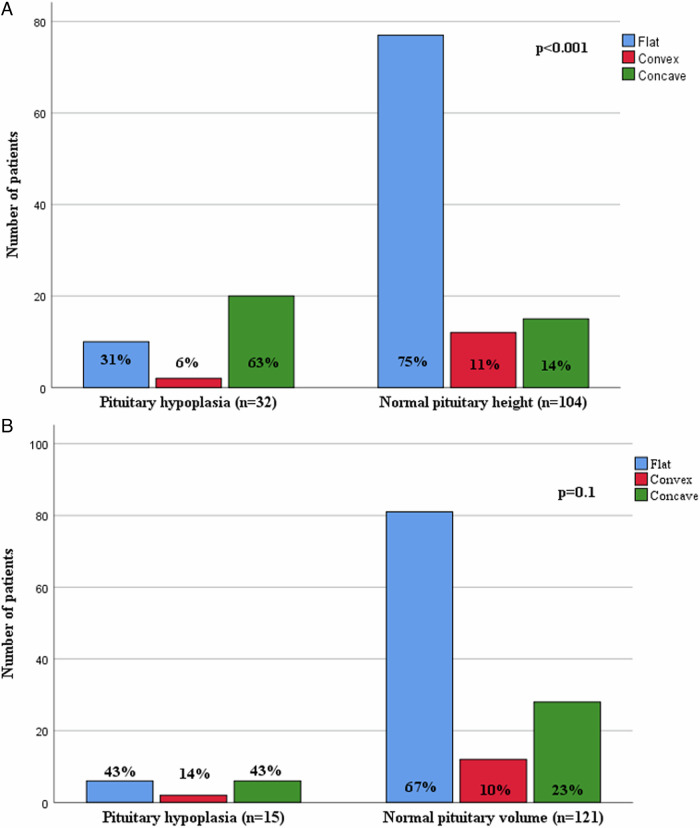
Features of the pituitary upper border of the patients with and without pituitary hypoplasia based on pituitary height (**A**) and pituitary volume (**B**)

Pituitary height and PV were significantly larger in patients with ISS compared to the patients with GHD (p = 0.03; p = 0.006) (Table 2). Both PH and PV were smaller in the prepubertal GH-deficient children compared to prepubertal children with ISS (p = 0.04; p = 0.03), however PV was alone smaller in pubertal GH-deficient children (p = 0.01). 72% of patients with ISS had flat, 5% had convex, and 23% had concave upper border, whereas 61% of GH-deficient patients had flat, 12% had convex and 27% had concave upper border (p = 0.6). While prepubertal children mostly tend to have flat upper border (70%), the frequency of convexity (11%) and concavity (29%) was slightly higher in pubertal ages, independently of gender (p = 0.3). The concavity rate was found to be significantly higher in patients with hypoplasia based on PH, regardless of whether it was based on CA, HA, or BA (p < 0.001). When hypoplasia was determined based on PV, the concavity ratio in patients with hypoplasia was found to be similar to that in patients without hypoplasia (Fig. 1).

**Table 2:** Pituitary measurements of patients with severe and partial growth hormone deficiency and idiopathic short stature

	Severe GHD (n = 57)	Partial GHD (n = 40)	ISS (n = 39)	*p*-value
Pituitary height, mm	4.4 ± 1.5	4.7 ± 1.4	5 ± 1.2	0.03
Pituitary width, mm	7.3 ± 1.5	7.5 ± 1.14	8 ± 1	0.09
Pituitary lenght, mm	12.2 ± 2	12.3 ± 1.6	12.8 ± 1.8	0.1
Pituitary volume, mm^3^	187 ± 89	209 ± 96	248 ± 107	0.006

*GHD* Growth hormone deficiency, *ISS* Idiopathic short stature

On the other hand, PV was found to be associated with more clinical findings including height SDS, HV before and after rGH therapy, stimulated GH, IGF-1 SDS/IGFBP-3 SDS compared with other unidimensional measurements (Table 3). We found that a closer relationship was revealed between PV and BA more than CA and HA. PH did not correlate with CA, a weak correlation was detected between PH and HA, and BA. Moreover, we found that PV was only significant predictor on GHD diagnosis in the model including BA, IGF-1 SDS, PH, PV, width, and lenght (OR:0.3, 95% Confidence interval(CI):0.08–0.9, p = 0.03).

**Table 3:** Correlations between pituitary measurements and clinical features

		Pituitary height	Pituitary width	Pituitary lenght	Pituitary volume
Chronological age	*r* *p*	0.12 0.14	0.04 0.6	0.35 < 0.001	0.28 0.001
Height age	*r* *p*	0.18 0.03	0.05 0.6	0.37 < 0.001	0.32 < 0.001
Bone age	*r* *p*	0.22 0.009	0.03 0.7	0.37 < 0.001	0.36 < 0.001
Height SDS	*r* *p*	0.02 0.8	0.03 0.6	−0.005 0.9	0.17 0.04
HV before GH treatment	*r* *p*	0.02 0.9	0.09 0.3	0.1 0.2	0,18 0.03
HV after GH treatment	*r* *p*	−0.16 0.07	0.05 0.5	0.13 0.1	−0.23 0.007
Height SD gain	*r* *p*	−0.01 0.8	−0.05 0.6	−0.1 0.2	−0.22 0.01
Stimulated GH	*r* *p*	0.26 0.002	0.05 0.5	0,02 0.9	0,26 0.002
IGF-1	*r* *p*	0.21 0.01	0.37 < 0.001	0.23 0.008	0.41 < 0.001
IGFBP-3	*r* *p*	0.08 0.3	0.33 < 0.001	0.26 0.003	0.26 0.003

*GH* Growth hormone, *HV* Height velocity

On the other hand, we investigated the most reliable method for determining pituitary hypoplasia on which basis, PH or PV, and according to which of CA, HA or BA. We analyzed the diagnostic accuracy of each method. BA-based PV tend to be able to discriminate ISS from GHD better than the other methods (Table 4).

**Table 4:** The frequency of pituitary hypoplasia in patients with GHD and ISS according to pituitary volume and pituitary height based on CA, HA or BA

	Pituitary hypoplasia rate		Sensitivity, %	Specificity,%	PPV, %	NPV, %
	GHD *n* (%)	ISS *n* (%)				
Pituitary volume						
Based on CA	21 (21)	3 (7)	22	92	88	32
Based on HA	10 (10)	1 (3)	10	97	90	30
Based on BA	13 (13)	1 (3)	17	98	94	39
Pituitary height						
Based on CA	44 (45)	10 (25)	45	74	81	35
Based on HA	26 (27)	7 (18)	27	82	78	31
Based on BA	25 (26)	7 (18)	26	82	78	30

*BA* Bone age, *CA* Chronological age, *GHD* Growth hormone deficiency, *HA* Height age, *ISS* Idiopathic short stature, *NPV* Negative predictive value, *PPV* Positive predictive value

On the basis of PV 21 patients (21%) with GHD and 3 patients (8%) with ISS had pituitary hypoplasia based on CA, 10 patients (10%) with GHD and 1 patient (3%) with ISS had pituitary hypoplasia based on HA, and 13 patients (13%) with GHD and 1 patient (3%) with ISS had pituitary hypoplasia based on BA.

On the basis of PH, the frequency of pituitary hypoplasia was higher. 44 patients (45%) with GHD and 10 patients (25%) with ISS had pituitary hypoplasia based on CA, 26 patients (27%) with GHD and 7 patients (18%) with ISS had pituitary hypoplasia based on HA, and 25 patients (26%) with GHD and 7 patients (18%) with ISS had pituitary hypoplasia based on BA.

We also investigated clinical characteristics of the GH-deficient patients with or without pituitary hypoplasia based on PV and according to CA, HA, or BA. Birth weight, pubertal grading, sex distribution, height SDS, mean doses and duration of rGH and IGFBP-3 SDS were similar in each group. When pituitary hypoplasia diagnosed based on PV-CA, stimulated GH was 5 ± 2.4 ng/mL in the patients with hypoplasia and 7 ± 2 ng/mL in the patients with normal PV (p < 0.001). Similarly IGF-1 SDS were found to be significantly lower in those with pituitary hypoplasia (−1.7 ± 1.1&-1 ± 1.2, p = 0.007). Stimulated GH was lower in the patients with pituitary hypoplasia based on PV-HA (6.8 ± 3.7 & 8.7 ± 4.4 ng/mL, p = 0.03). All clinical features were similar in the groups with and without hypoplasia when evaluated according to BA. Pituitary hypoplasia, based on both PH and PV, was detected at a similar rate in patients with severe and partial GHD.

Also clinical characteristics of GH-deficient patients with and without hypoplasia, that diagnosed based on PH and according to CA, HA, or BA were compared. Birth weight, pubertal grading, sex distribution, height SDS, stimulated GH, mean doses and duration of rGH and IGFBP-3 SDS were similar in each group. The mean IGF-1 SDS was significantly higher in the patients with normal PH compared with the patients with pituitary hypoplasia based on CA (−1.8 ± 1.5&-1.2 ± 1.2, p = 0.02). However in the groups formed according to HA or BA, there was no difference between clinical characteristics of patients with and without hypoplasia.

On binary logistic regression, lower stimulated GH and IGF-1 SDS were independently associated with increased likelihood of having pituitary hypoplasia based on PV-CA (1.26 95%CI:1.12–1.42, p < 0.001 and 1.5, 95%CI:1.1–2.1, p = 0.014).

We also investigated effect of method for diagnosing hypoplasia on the prognosis of GHD. After rGH therapy, GH-deficient patients with pituitary hypoplasia based on PV-CA were found to have significantly higher height SD gain compared with the patients with normal PV (1.6 ± 1, 0.8 ± 0.6, p = 0.03). When pituitary hypoplasia was diagnosed according to PH-CA, rGH responses were found to be similar in the groups. After multiple linear regression analysis, pubertal status and CA at presentation were significant predictors for height SD gain, afterwards, decrease in PV resulting a better rGH response (2.73 + -0.55*PV 95%CI (−0.85)-(−0.24), p = 0.001).

During a median of 3 (1.2–4.8) years of follow-up, only ACTH deficiency was added to GHD in six GH-deficient patients. One of these had normal PV and five of these had pituitary hypoplasia based on PV-BA, whereas two patients with ACTH deficiency had normal PV, and four of those had pituitary hypoplasia diagnosed according to PV-CA. After multiple logistic regression analysis, IGF-1 SDS (−4.2 95%CI(1.2–14), p = 0.02) and PV (−5.2 95%CI (1.08–25) p = 0.04) were the only predictors for ACTH deficiency. Also we found severity of GHD tend to be a risk factor for developing MPHD (−0.45 95%CI 0.39–1.03, p = 0.06).

## Discussion

We found that PH and PV were significantly smaller in GH-deficient patients compared to patients with ISS, consistently with the previous reports [2, 3]. However, during pubertal period, only PV was found to be different between patients with GHD and ISS. The fact that only PV was significantly lower in the patients with GHD during both childhood and adolescence, may indicate that diagnosis of pituitary hypoplasia based on PV is more reliable throughout childhood. There are conflicting results in the literature. While some of studies recommend using PH for diagnosis, recently there are increasing number of studies that recommend using PV as a basis [2, 4, 6, 10, 11]. The common view of majority of the reports, recommending PG size should be evalutaed based on PV, is PG has various morphologies, so it is not always accurate to assess of PG size on one dimension.

In our study, we obtained some new findings about the PG that were not well known. We found 2–7 fold increase in the frequency of pituitary hypoplasia when diagnosed based on PH compared to the method based on PV. We can explain this discrepancy as 25% of all patients had markedly concave upper border of the PG. Most of them (75%) were patients with GHD. Similarly, Dumrongpisutikul et al. reported concave upper border of PG was observed significantly more often among GHD patients (16%) compared to healthy subjects [12]. On the other hand, in the current study, concavity rate was higher in the patients with pituitary hypoplasia diagnosed based on PH, whereas concavity rate was similar when hypoplasia diagnosed based on PV, leading PH might be incorrectly interpreted as shorter. So, reliability of assessment of pituitary size based on PH may lead to debate. So PV might be more reliable in assessing pituitary size than PH alone, especially in the patients with GHD.

All PG measurements were related to IGF-1. But only two of PH and PV were related to GH secretion consistently with the literature [10, 13, 14]. On binary logistic regression, lower stimulated GH and IGF-1 SDS were independently associated with increased likelihood of having pituitary hypoplasia. And stimulated GH was lower in the patients with hypoplasia based on PV-CA consistently previous reports [15]. As well as, previously, it was reported that children with lower stimulated GH had a higher prevalence of abnormal pituitary imaging [15, 16]. Maghnie et al. recommended 3 ng/mL and Oren et al. recommended 4 ng/mL for the stimulated GH cut-off value to predict pituitary abnormality. Unlike previous reports, frequency of pituitary hypoplasia based on PV-BA was similar, in those with stimulated GH of below 7 ng/mL (12%), and of 7–10 ng/mL (15%). Likewise, in a previous study including nearly 500 patients, both lower stimulated GH and baseline IGF-1 concentrations were independently associated with increased odds of a pathogenic MRI, but differently, pituitary abnormalities were reported uncommon, particularly in those with stimulated GH of 7.0–10 ng/mL (1.5%) [17]. In a recently published review by Yuen et al. it was suggested that any pituitary abnormalities might be helpful in facilitating decision on rGH therapy in children with stimulated GH of 7–10 ng/mL [18]. Similarly, in our study, if patients with partial GHD had pituitary hypoplasia (based on PV-BA), they tend to have more height SD increment compared to ones with normal PV (1.1 ± 0.9 & 0.7 ± 0.6, p = 0.1).

While planning our study another question that we aim to clarify is whether PG size should be evaluated according to CA, HA, or BA for defining pituitary hypoplasia, especially in the children with short stature that might have delayed HA and BA. In almost all previous studies, PG size was evaluated based on CA. Hilczer et al. evaluated the PG size respect to CA and HA [19]. They found that pituitary hypoplasia based on HA was more closely related to clinical findings, and was associated with more severe GHD. To our knowledge, it was the first study that evaluated PG size based on HA. HA and BA delay was more than 2 years compared to CA in our study. We found that PH did not correlate with CA, and a weak correlation was detected between PH and HA, and BA. This finding was inconsistent with previous studies reporting that PH increases with CA [20]. But there was a positive correlation between CA and PV in consistent with previous researches [8, 21]. In addition to the previous reports we found a closer relationship between both PH and PV, and BA rather than CA or HA.

Similarly Wu et al. reported BA was more significant predictor for PV than CA [22]. It might be considered that BA is closely associated with sex steroids, in this way Wong et al. showed pubertal stage had predictive power beyond age [23]. Especially considering close relationship between increasing sex steroids and PG size during puberty, evaluation of PG size according to BA becomes important at puberty.

When pituitary hypoplasia was diagnosed according to CA based on both PH and PV, we found pituitary hypoplasia rate 2–3 fold higher in the patients with GHD and ISS. However, previous studies have been inconlusive, reporting a wide variation in the prevelance of pituitary hypoplasia ranging from 7% to 84% in the GH-deficient patients, and 8–35% in the patients with ISS [1, 11, 24–26]. In a study, more than 15,000 children with GHD were evaluated, the frequency of hypoplasia was reported as 7.8% [1]. Despite the low sensitivity, we detected pituitary hypoplasia in 13% of patients with GHD by PV-BA-based diagnosis. On the other hand, based on PV-BA, pituitary hypoplasia was observed in 3% of patients with ISS. To our knowledge, this is the lowest frequency of pituitary hypoplasia in the patients with ISS in the literature. PV-BA based diagnosis has the higher specificity, positive and negative predictive value. We believe that evaluating pituitary hypoplasia based on CA might have caused the increased frequency of hypoplasia. In particular, in short children that may have HA and BA delay, determination of the hypoplasia based on BA may prevent false-positive results. So, we might suggest the clinicians to define pituitary hypoplasia based on BA instead of CA or HA. However, there was still one patient with ISS who had pituitary hypoplasia. Although there are no clear data, patients with ISS are not expected to have any pituitary abnormalities. However, there are several studies reported pituitary hypoplasia in the patients with ISS [1]. In a previous study, it was reported that patients with ISS had smaller PV than healthy ones [2]. They showed that if patients have small PG, even if they had normal stimulated GH, small PG can not achieve increased GH secretion during puberty. Consistent with this report, in our study while most of the clinical features were similar between severe GHD and partial GHD, clinical features were clearly different between patients with and without pituitary hypoplasia based on PV. As a result, we may suggest PG size might be more reliable than GH stimulation tests for diagnosing GHD and making treatment decision. Similarly, there are reports showing PG size is important in diagnosing GHD [10]. Although BA was more related to PG size, the clinical features of patients with and without hypoplasia could be distinguished, when pituitary hypoplasia was diagnosed based on PV-CA. This was likely due to small number of patients with pituitary hypoplasia based on PV-HA and PV-BA. We believe that further analysis with more participitants is warrented.

In the current study, during 3-years follow-up, we observed 0.8 SD higher height SD gain in the patients with pituitary hypoplasia, after rGH therapy compared with the patients with normal PV. When pituitary hypoplasia was diagnosed according to PH, rGH responses were found to be similar in the groups. 15% of the patients with GHD had poor GH response, 25% of poor responders had severe, whereas 75% of those had partial GHD. But, higher GH secretion was not associated with poor GH response, on binary logistic regression. We found reduced PV had better response to rGH. Likewise, previous reports demonstrated that pituitary abnormalities including pituitary hypoplasia was a significant determinant of rGH response, smaller PG was related better rGH response, and was more accurate than stimulated GH [14, 27]. Recently it was reported that PG size was the most important characteristic affecting height gain [28, 29]. In all of the reports, pituitary hypoplasia was diagnosed based on PH or PV according to CA. Conversely, several reports have revealed that puberty, age and height SDS at presentation were related to rGH response whereas, there was no relationship between PG size and rGH response [30, 31]. Based on our study, the fact that rGH response was related to PV, may indicate PV can be an important diagnostic and prognostic tool for GHD. On the other hand, the fact that most of the poor responders have partial GHD, shows that GH secretion also affects prognosis. Moreover, partial GH-deficient patients with pituitary hypoplasia based on PV-CA, had better rGH response. So, we can suggest pituitary hypoplasia is more accurate for predicting prognosis more than GH stimulation tests.

ACTH deficiency was developed 6% of GHD patients, consistently with the literature [32]. Maghnie et al. suggested pituitary MRI findings in the patients with GHD might be the most important criterion rather than GH stimulating testing for developing MPHD [16]. Likewise, after logistic regression analysis, IGF-1 SDS and PV were the only predictors for MPHD. Also we found severity of GHD tend to be a risk factor for developing MPHD. Although Bozzola et al. reported that isolated pituitary hypoplasia does not significantly contribute to developing MPHD, in the absence of additional anatomical defects such as pituitary stalk agenesis [31]. Pituitary hypoplasia has been reported as an important risk factor in development of MPHD in the patients with isolated GHD [4, 16, 33, 34]. ACTH deficiency was found to be 5-fold higher in patients with pituitary hypoplasia diagnosed based on PV-BA than in patients without hypoplasia, whereas it was only 2-fold higher when hypoplasia was determined based on PV-CA. Similarly, in a previous study in which pituitary hypoplasia was defined based on PH-CA, it was shown that, pituitary hypoplasia rate was found to be only 2-fold higher in patients with MPHD compared with the patients with isolated GHD [4]. We can suggest patients with pituitary hypoplasia defined according to PV-BA will need closely follow up owing to the risk of MPHD.

Our study is the first in which the relationship of each diagnostic method used to define pituitary hypoplasia with clinical manifestations was examined in detail. There might be racial differences in pituitary size [35]. In our study, comparison of the PG size with Turkish normal data is one of the features that strengthens the study. Also, our study has some limitations. The main limitation was small sample size. The other limitation was having any patient reached the final height. So, we could not evaluate the effect of pituitary hypoplasia on the permanent GHD, long-term response to rGH therapy. Additionally we evaluated PV by using mathematical formulae for ellipsoid. However, PG may not be an ellipsoid, because the morphology of the PG changes continuously throughout childhood [36]. The effects of sex steroids on height and BA are well known. One of the limitations of the study was the lack of gonadotropin and sex steroid levels.

## Conclusion

PV was a significant predictor for response to rGH therapy and the risk of developing MPHD in the patients with GHD. PV based on BA can contribute to diagnose GHD, and to be able to discriminate ISS from GHD. So, we may suggest that pituitary hypoplasia should be diagnosed based on BA by using PV. But it should be kept in mind that there might be still misdiagnosed patients by this method.
